# A Critical Review on the Economically Feasible and Sustainable Poly(3-Hydroxybutyrate-*co*-3-hydroxyvalerate) Production from Alkyl Alcohols

**DOI:** 10.3390/polym14040670

**Published:** 2022-02-10

**Authors:** Hau Seung Jeremy Wong, Kesaven Bhubalan, Al-Ashraf Abdullah Amirul

**Affiliations:** 1 School of Biological Sciences, Universiti Sains Malaysia, Gelugor 11800, Penang, Malaysia; jeremywong@student.usm.my; 2 Centre for Chemical Biology, Universiti Sains Malaysia, Bayan Lepas 11900, Penang, Malaysia; 3 Eco-Innovation Research Interest Group, Faculty of Science and Marine Environment, Universiti Malaysia Terengganu, Kuala Nerus 21030, Terengganu, Malaysia; kesaven@umt.edu.my; 4 Institute of Marine Biotechnology, Universiti Malaysia Terengganu, Kuala Nerus 21030, Terengganu, Malaysia

**Keywords:** 1-pentanol, 1-propanol, 3-hydroxyvalerate precursor, alkyl alcohol tolerance, biosynthesis, oxo synthesis, polyhydroxyalkanoates, poly(3-hydroxybutyrate-*co*-3-hydroxyvalerate), propionic acid, valeric acid

## Abstract

Poly(3-hydroxybutyrate-*co*-3-hydroxyvalerate) (P(3HB-*co*-3HV)) is the most studied short-chain-length polyhydroxyalkanoates (PHA) with high application importance in various fields. The domination of high-cost propionate and valerate over other 3-hydroxyvalerate (3HV) precursors owing to their wide preference among PHA-producing bacteria has hindered the development of diverse production processes. As alkyl alcohols are mainly produced from inexpensive starting materials through oxo synthesis, they contribute a cost-effective advantage over propionate and valerate. Moreover, alkyl alcohols can be biosynthesized from natural substrates and organic wastes. Despite their great potential, their toxicity to most PHA-producing bacteria has been the major drawback for their wide implementation as 3HV precursors for decades. Although the standard PHA-producing bacteria *Cupriavidus necator* showed promising alcohol tolerance, the 3HV yield was discouraging. Continuous discovery of alkyl alcohols-utilizing PHA-producing bacteria has enabled broader choices in 3HV precursor selection for diverse P(3HB-*co*-3HV) production processes with higher economic feasibility. Besides continuous effort in searching for promising wild-type strains, genetic engineering to construct promising recombinant strains based on the understanding of the mechanisms involved in alkyl alcohols toxicity and tolerance is an alternative approach. However, more studies are required for techno-economic assessment to analyze the economic performance of alkyl alcohol-based production compared to that of organic acids.

## 1. General Overview

Polyhydroxyalkanoates (PHA) are emerging as the next generation plastics owing to their plastic-like properties, renewability, biodegradability, and biocompatibility [1]. PHA are accumulated by bacteria under carbon excess but nitrogen-limiting conditions and stored as a reserved energy source in the form of single or multiple granules in the cytoplasm [2]. PHA have gained much industrial interest in the last few decades due to their potential as substitutes for conventional plastics, and various fermentation strategies have been developed to establish microbial PHA production for commercialization. Poly(3-hydroxybutyrate-*co*-3-hydroxyvalerate) (P(3HB-*co*-3HV)) is the most studied PHA copolymer with mechanical properties comparable to that of polypropylene. The 3-hydroxyvalerate (3HV) monomer provides elastomeric property to the copolymer, enabling broader application compared to the homopolymer poly(3-hydroxybutyrate) (P(3HB)) [3]. The improvement in mechanical properties has paved the way for it to be established for medical, tissue engineering, aquacultural, agricultural, and commodity applications. The commercialization of P(3HB) and P(3HB-*co*-3HV) started in the 1970s by Imperial Chemical Industries, U.K., and Chemie Linz AG, Austria [4]. Currently, P(3HB) and P(3HB-*co*-3HV) are commercialized by TianAn Biopolymer, China, and Sigma-Aldrich, USA.

Commercialization of PHA is hampered by its high production cost, majorly due to the cost of the carbon feedstock used in microbial fermentation. Over recent decades, various industrial wastes were explored as alternative carbon sources, and numerous mitigation strategies were taken to establish microbial production of P(3HB-*co*-3HV) with high economic feasibility at a commercial scale. Bioconversion of unrelated carbon sources into P(3HB-*co*-3HV) was attempted, but metabolic engineering strategies are generally required to promote precursor-independent pathways to synthesis P(3HB-*co*-3HV), with exceptions for wild types *Nocardia* or *Rhodococcus* that can generate propionyl-CoA endogenously from a single carbon source [5,6,7,8,9]. Owing to the relatively simpler practical requirement, P(3HB-*co*-3HV) production from related carbon source(s) remains competitive. Although the employment of wastes contributes to higher economic feasibility, P(3HB-*co*-3HV) production from a single carbon source has low practicability due to the composition inconsistency of raw components for 3HV formation [3].

The most common way to incorporate 3HV monomers is by employing a precursor carbon source as a co-substrate along with the main carbon source that contributes to the 3-hydroxybutyrate (3HB) monomer. Precursor carbon sources such as organic acids, alcohols, or some amino acids were studied thoroughly to clarify the metabolic pathways involved and to search for promising precursors of greater potential. Organic acids, especially propionic acid, valeric acid, and their respective salts, are the standard 3HV precursors owing to their wide acceptance among PHA-producing bacteria. However, organic acids can only be added in low concentrations due to their high toxicity to the bacteria, and their high substrate cost causes lower profitability. Although levulinic acid is way more cost-effective than propionic acid and valeric acid, it seems to be a privilege for *Cupriavidus necator,* and the production mechanism is yet to be clarified [10,11]. Although some amino acids such as threonine, valine, and isoleucine could be employed as 3HV precursors, metabolic engineering of the amino acid biosynthetic pathways is required to convert amino acids into propionyl-CoA, which is essential for 3HV formation. The rare occurrence of alcohols-utilizing ability among PHA-producing bacteria hinders the employment of alkyl alcohols as 3HV precursors despite their potential as cost-effective substitutes for organic acids [3]. In addition to the merit in lowering the substrate cost, naturally occurring carbon sources such as glucose and glycerol or organic wastes can be converted by microorganisms into alkyl alcohols, thus are promising as cost-effective and sustainable bioresources for P(3HB-*co*-3HV) production [12].

*C. necator* is the standard PHA-producing bacterium well-known with its wide substrate acceptance range, including alcohols and mercury. Nevertheless, its capability to convert alcohols into PHA is substandard. The 3HV yield from 1-propanol is low despite its high tolerance toward 1-propanol, and the employment of 1-pentanol results in a remarkably high reduction in *C. necator* cell biomass and PHA content [10,13]. Owing to the economic advantage over organic acids, the employment of alcohols as the 3HV precursors for P(3HB-*co*-3HV) production was attempted for various bacteria. Interestingly, P(3HB-*co*-3HV)-producing bacteria favoring alcohols as 3HV precursors are emerging since the last decade. Since the discovery of *Paracoccus denitrificans* ATCC 17741 with the capability to convert 1-pentanol into 3HV in 1996, various alkyl alcohol-tolerant PHA-producing bacteria were discovered continually whereby several of them depicted promising 3HV yield [14].

This critical review condenses the production of P(3HB-*co*-3HV) from alkyl alcohols and the promising potential of alkyl alcohols as cost-effective 3HV precursors to go beyond the bottleneck in precursors selection that is limited to organic acids. The properties and applications of P(3HB-*co*-3HV) are also discussed. The bioconversion pathways of 1-propanol and 1-pentanol into 3HV with respect to propionic acid and valeric acid are visualized, and the performance of discovered alkyl alcohol-tolerant PHA-producing bacteria is highlighted. Oxo synthesis and biosynthesis of 1-propanol and 1-pentanol from natural substrates as well as organic wastes were described. Furthermore, the mode of action of alkyl alcohols on bacterial proteins and the bacterial mechanisms involved in response to alcoholic stress are also discussed. The strategies for wide implementation of alkyl alcohols for P(3HB-*co*-3HV) production and the challenges ahead are highlighted as well to comment on the potential of alkyl alcohols as the next generation 3HV precursors.

## 2. P(3HB-*co*-3HV) Properties and Applications

P(3HB) is a relatively stiff and brittle polyester with poor elongation at break [15]. It is a fragile material, and its mechanical properties deteriorate with time due to secondary crystallization accompanied by aging at room temperature, which is the major cause of its brittleness [16]. Although the lack of elasticity causes a drawback in its application as packaging materials, its high mechanical properties are applicable as bone tissues aid in supporting body weight. P(3HB) facilitates reconstructive osteogenesis. P(3HB) and its biocomposite incorporated with 20 wt% hydroxyapatite, which makes up 65–70% of the bone matrix, show pronounced osteoplastic properties owing to their slow degradation that corresponds to the growth of new bones. Powdered P(3HB) and P(3HB)/tienam are excellent antibacterial bone filling materials that contribute to 1-fold lower growth and complete growth inhibition of *Staphylococcus aureus* post surgery, respectively [17].

**Figure 1:**
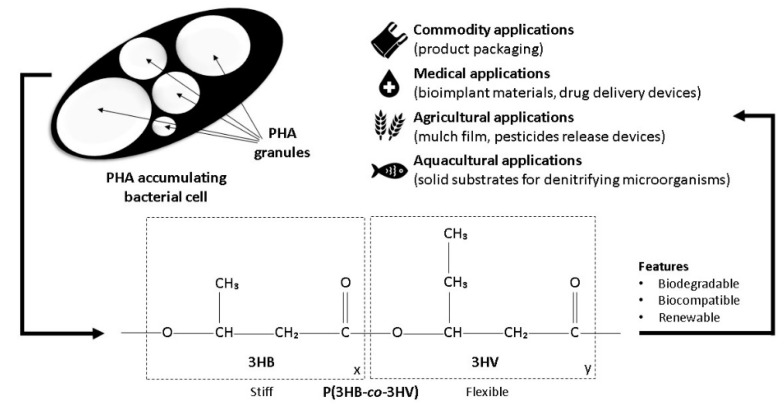
Microbial PHA granule, P(3HB-*co*-3HV) structure, and applications.

The incorporation of the C_5_ 3HV monomer into P(3HB) results in P(3HB-*co*-3HV) with decreased crystallinity, thus leading to decreased stiffness, decreased brittleness, and enhanced biodegradability compared to that of P(3HB) [18]. The properties of P(3HB-*co*-3HV) are dependent on the ratio of the two monomers where the 3HB monomer contributes stiffness, and the 3HV monomer contributes flexibility to the copolymer. The composition of the 3HV monomer determines the defection of the P(3HB) lamellae crystals, leading to the disruption of its crystallinity and resulting in improved polymer flexibility (Figure 1) [19]. The lower degree of crystallinity and melting point of P(3HB-*co*-3HV) lead to a higher degradation rate that is directly proportional to the molar fraction of 3HV of the copolymer compared to that of P(3HB) [18]. The 3HV fraction contributes to a greater amorphous region for enzymatic attacks that leads to enhanced and adjustable biodegradability for applications such as implants for bone support, stents for artery support in angioplasty, and drug delivery carriers. Although P(3HB-*co*-3HV) has a 2-fold lower maximum water permeability than poly(lactic acid) which is another biodegradable aliphatic polyester of great biotechnological importance, causing lower hydrolytic degradation due to lower water uptake, the degradation rate of P(3HB-*co*-3HV)-based biomedical devices are adjustable with molar fraction of 3HV [18,20,21]. Hydrophilic poly(ethylene glycol) and monomethoxy poly(ethylene glycol) can also be incorporated into P(3HB-*co*-3HV) to form nanoparticles with a hydrophilic outer layer and a hydrophobic inner layer for improved chemical functionalization and compatibility with therapeutic drugs besides benefiting drug release control [22,23,24]. Incorporation of other desired properties for biomedical applications can also be achieved (Table 1).

P(3HB-*co*-3HV) is a potential substitute for petroleum-based plastic packaging material as it possesses high water and aroma (limonene and linalool) barrier properties while having comparable thermal and mechanical properties to that of polypropylene (PP) and low-density polyethylene (LDPE) [15]. As PP and LDPE are applied extensively for packaging and consumables, which are highly disposable, the substitution with P(3HB-*co*-3HV) can contribute to reduced stable solid waste creation of petroleum-based plastics [47,48]. Unlike the augmented cytotoxicity by higher 3HV molar fraction, lower 3HV molar fraction causes high stereoregularity, slow crystallization rate, formation of large size spherulites, and secondary crystallization that are discouraging for packaging purposes [24,47,48]. Poly(butylene succinate), poly(butylene adipate-*co*-terephthalate), natural rubber, or other polymers with plasticizer or toughness properties can be incorporated to overcome the limitations and extend its application as packaging materials (Table 1).

Moreover, PHA-based mulch films are potential substitutes for conventional plastic mulch films. Mulching increases crops productivity, increases horticulture products, prevents water evaporation from the soil, prevents soil erosion, reduces water consumption, and controls weeds [49]. PHA-based mulch films overcome the environmental problems caused by the post-consumption of plastic mulch films made from LDPE, linear low-density polyethylene (LLDPE), and high-density polyethylene (HDPE) due to their poor degradability [50]. Moreover, the physicochemical properties of P(3HB-*co*-3HV) enable the controlled release of herbicides and insecticides. Herbicides and insecticides can be integrated into P(3HB-*co*-3HV)-containing pellets and sown along the plantation to be released upon degradation from the pellets depending on the level of pest activity [51,52].

On the other hand, endogenous P(3HB-*co*-3HV) acts as the electron donor for the denitrification of wastewater in the aquaculture industry. Biomass with PHA-accumulating ability, generally P(3HB) and poly(3-hydroxyvalerate) (P(3HV)), from activated sludge, is employed to remove resulting ammonia from fish excretion and dead animal bodies in circulating water. Unlike the conventional techniques that involve the addition of acetate and ethanol to promote microbial activity, the biomass is precultured for PHA accumulation. The endogenous PHA is used for denitrification that accurately couples with slow metabolic activity in the absence of exogenous carbon source and in the presence of nitrogen [53,54]. The exclusion of volatile fatty acids feeding during the denitrification process prevents the contamination with the dissolved organic carbon that lowers the effluent water quality, and the employment of endogenous PHA is more cost-effective compared to feeding extracted PHA to denitrifying bacteria [55].

## 3. Bioconversion of Alkyl Alcohols and Organic Acids into P(3HB-*co*-3HV)

**Figure 2:**
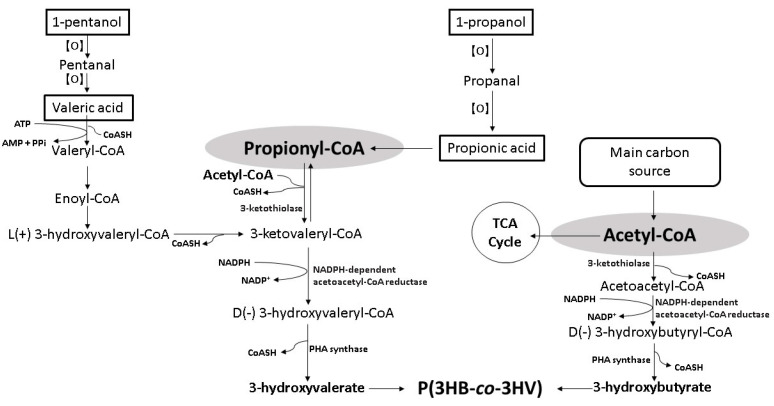
Schematic bioconversion pathway of organic acids and alkyl alcohols into 3HV [14,57,58,59,60].

The conversion of organic acid into 3HV starts with *β*-oxidation, where propionic acid (C3) is converted into propionyl-CoA, whereas valeric acid (C5) is converted into propionyl-CoA and acetyl-CoA, respectively [56]. The 3HV monomer is formed from the resulting propionyl-CoA couples with acetyl-CoA and is polymerized to P(3HB-*co*-3HV) copolymer with the 3HB monomer. The 3HB monomer is formed from the resulting acetyl-CoA provided majorly by the main carbon source such as oils or sugars (Figure 2) [14,57,58,59,60].

The employment of alkyl alcohols as 3HV precursors is limited to odd carbon number primary alcohols. Primary alcohols are oxidized to aldehydes that can be further oxidized more easily to their respective carboxylic acids. The oxidation processes can occur chemically with the presence of oxidizing agents or biologically with the presence of alcohol dehydrogenase and aldehyde dehydrogenase [61]. Oxidation of secondary alcohols liberates ketones with no further oxidation due to the oxidatively stable nature of ketones [62,63]. Odd carbon number primary alcohols such as 1-propanol or 1-pentanol are oxidized to 1-propanal and 1-pentanal that further oxidized to propanoic acid and valeric acid, respectively. The resulting propionic acid or valeric acid enters *β*-oxidation to liberate propionyl-CoA for 3HV formation (Figure 2) [14,57,58,59,60].

Although levulinic acid is a cost-effective 3HV precursor, the catabolic pathway involved is undetermined. Generally, levulinic acid catabolism releases intermediates that are converted via *β*-oxidation to release acetyl-CoA and propionyl-CoA for P(3HB-*co*-3HV) biosynthesis [64]. Bacteria capable of using levulinic acid as the 3HV precursor are rare and are mainly *C. necator*, with the exception of *Burkholderis* sp. IS-01 and *Hydrogenophaga pseudoflava* DSM 1034 [10,11,65,66,67,68]. *C. necator* KHB-8862 and *H. pseudoflava* DSM 1034 are two promising strains reported with a high 3HV yield of 0.50 and 1.00 g/g, respectively. However, other studies reported low PHA content and 3HV yield (Table 2).

## 4. Techno-Economic and Sustainability Assessment

**Figure 3:**
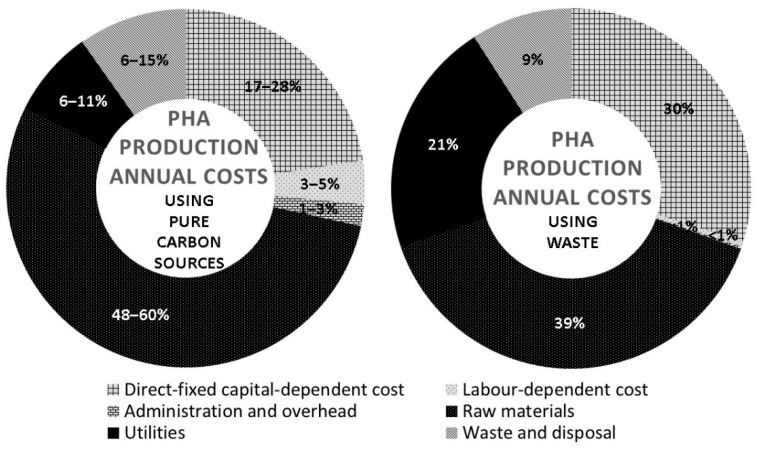
Techno-economic analysis on PHA production annual costs using pure carbon sources and wastes [93,95].

The annual operating costs in PHA production generally include the direct fixed capital-dependent items, labor-dependent items, administration, and overhead expenses, raw materials, utilities, and downstream processing such as waste management. According to the techno-economic analysis conducted by Choi and Lee (1999) for various pure carbon sources, the substrate cost accounted for 48%–60% of the total costs (Figure 3) [93]. After excluding the trace elements, which are essentials, pure carbon sources that possess high nutritional value such as glucose, glycerol, starch, methane, oils, and volatile fatty acids are commercial products, and their employment leads to higher substrate cost compared to that of industrial or domestic wastes. Due to higher economic advantage and increasing emphasis on sustainability, the employment of wastes as carbon sources is widely attempted. Theoretically, substituting pure substrates with wastes contributes to a huge reduction in raw material expenses. However, pretreatments are needed for certain wastes to remove impurities and toxins or to adjust pH [94]. Pretreatments impose additional costs whereby extra chemicals or equipment are necessary with possible individual optimization. Bhattacharyya and co-workers (2015) reported decreased raw material cost to 39% with the employment of wheat stillage, but the utilities cost increased to 21% as compared to that reported by Choi and Lee (1999) (Figure 3) [93,95].

As opposed to main carbon sources, where numerous studies have been conducted on various wastes, employing wastes as 3HV precursors is not practical due to the composition inconsistency [95]. Due to the necessity of propionyl-CoA for 3HV formation, sole reliance on wastes results in the narrow choice to those with propionate or valerate related components; thus, in most cases, a 3HV precursor is still required to achieve sufficient 3HV fraction for the copolymer to be practically useful [64,95]. This leads to increased raw material cost as propionic acid and valeric acid, which are widely preferred by PHA-producing bacteria, are high-cost precursors (Table 2). The potential of 1-propanol and 1-pentanol as alternatives for propionic acid and valeric acid is well-known but lack practicality due to its high toxicity to the majority of bacteria. Since 1996, several PHA-producing bacteria from different genera have been reported to use 1-propanol or/and 1-pentanol as 3HV precursors (Table 2). The emergence of these bacteria bypasses the bottleneck of precursor dominance by organic acids and enables further innovation in fermentation strategies to develop economically feasible and sustainable production processes. Furthermore, 1-propanol and 1-pentanol are manufactured through well-established oxo synthesis and can be biosynthesized by bacteria from sustainable carbon sources such as glucose, glycerol, and organic wastes, which are abundant in nature.

## 5. Oxo Synthesis of Alkyl Alcohols

**Figure 4:**
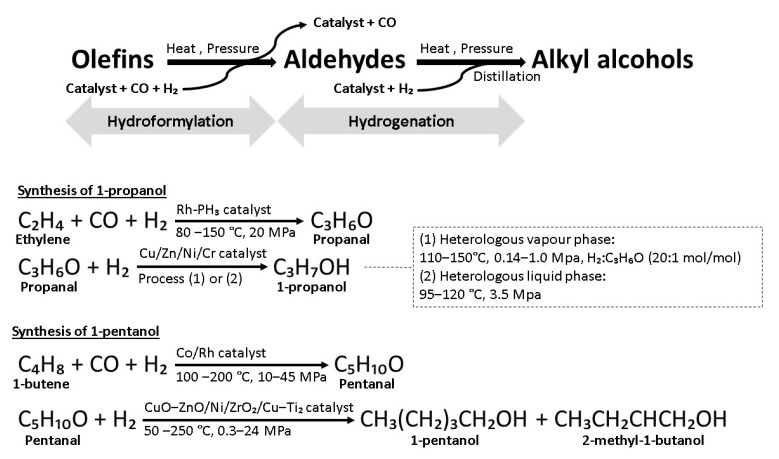
Oxo synthesis of alkyl alcohols [62,97,99].

Oxo synthesis is an established process for the manufacture of alkyl alcohols at an industrial scale with simple operational requirements and low specificity in raw materials, including branched-chain, long-chain, and cyclic olefins [96,97]. It is thoroughly investigated for the production of a wide variety of industrial chemicals. The synthesis involves hydroformylation to convert olefins (also known as alkenes) into aldehydes to be further converted into alcohols through hydrogenation. Homogeneous catalysts are employed in hydroformylation, while heterogenous catalysts are employed in hydrogenation for reaction induction. Generally, these reactions are carried out in separate reactors where the resulting aldehydes from the primary reactor are transferred into the second reactor to be hydrogenated. Catalysts and carbon monoxide in the primary reactor are removed either by decobalting or been recycled back to the primary reactor to prevent entry into the second reactor as a precautious measure to extend the shelf life of hydrogenation catalysts. Recycling the catalysts contributes to high economic feasibility as high-cost catalysts such as rhodium-based catalysts can be reused for subsequent batches. However, an 8–55% decrease in catalyst yield after repeated recycling is expected [98]. The resulting alcohols are purified from the mixture via distillation (Figure 4) [97].

### 5.1. 1-Propanol

Oxo synthesis of 1-propanol begins with the rhodium-catalyzed hydroformylation of ethylene (also known as ethene) to propanal with the aid of rhodium–triphenylphosphine catalysts. The resulting 1-propanal is distilled from the catalyst-containing solution, and carbon monoxide is removed. Hydrogenation can be carried out in either the heterogenous vapor phase or the heterogenous liquid phase. Heterogeneous vapor phase hydrogenation takes place at 110–150 °C and 0.14–1.00 MPa with the aid of copper, zinc, nickel, and chromium catalysts supported on alumina (CAS:1344-28-1) or kieselguhr (CAS:91053-39-3) [62]. Heat is removed either by an external heat exchange device or an internal cooler [100]. This process produces impurities such as dipropyl ether, ethane, and propyl propionate. Selectivity enhancers such as alkali or transition metals are added to reduce the formation of esters, while an additional 1%–10% water could suppress the formation of ether [62,101]. Propyl propionate is separated from the product mixture and hydrogenolyzed with the aid of reduced CuO–ZnO catalysts at 75–300 °C and 9.8 kPa–9.8 MPa to produce 1-propanol as the major product [62]. Heterologous liquid phase hydrogenation involved nickel or copper catalysts at a lower temperature of 95–120 °C and a higher pressure of 3.5 MPa. Crude 1-propanol is purified via distillation with the aid of an azeotroping agent such as dipropyl ether or cyclo-hexane to remove water for highly pure 1-propanol yield (>99%) (Figure 4) [62,102].

### 5.2. 1-Pentanol

Oxo synthesis of 1-pentanol begins with hydroformylation of 1-butene. Subsequent hydrogenation yields two C_5_ products that are 1-pentanol and 2-methyl-1-butanol. For cobalt-catalyzed hydroformylation, the ratio of the product is 7:3 (1-pentanol:2-methyl-1-butanol) after subsequent hydrogenation. When rhodium–triphenylphosphane is employed instead, a higher yield of 1-pentanol is achieved with a 9:1 (1-pentanol:2-methyl-1-butanol) ratio (Figure 4) [99].

## 6. Biosynthesis of 1-Propanol and 1-Pentanol by Wild-Type Bacteria

### 6.1. The Wood–Werkman Pathway in Propionibacteria

**Figure 5:**
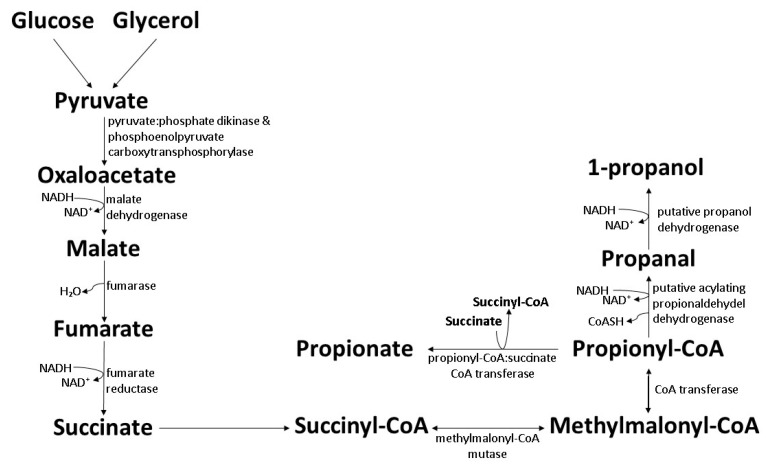
Biosynthesis of 1-propanol by wild-type *Propionibacteria* through the Wood–Werkman pathway [103,104].

Biosynthesis of 1-propanol by wild-type bacteria is inefficient as 1-propanol is synthesized as a byproduct through propionic acid synthesis processes. *Propionibacteria* such as *Propionibacterium acidipropionici* and *Propionibacterium freudenreichii* are able to produce 1-propanol through the Wood–Werkman pathway (also known as the dicarboxylic pathway, or the methylmalonyl-CoA pathway). The synthesis process requires an anaerobic condition where the carbon source is converted into pyruvate and enters the Wood–Werkman pathway to produce propionic acid as the main product [103,104]. The 1-propanol yield reported was in the range of 0.04–0.14 mol/mol, equivalent to 0.6–1.8 g/L. The 1-propanol production was found to be higher when glycerol was employed, compared to glucose [105,106]. The precise processes involved in 1-propanol formation are undetermined but could probably be by two-step reduction from propionyl-CoA to 1-propanol aided by acylating propionaldehyde dehydrogenase and propanol dehydrogenase (Figure 5) [107].

### 6.2. The Acrylate Pathway in Clostridium

**Figure 6:**
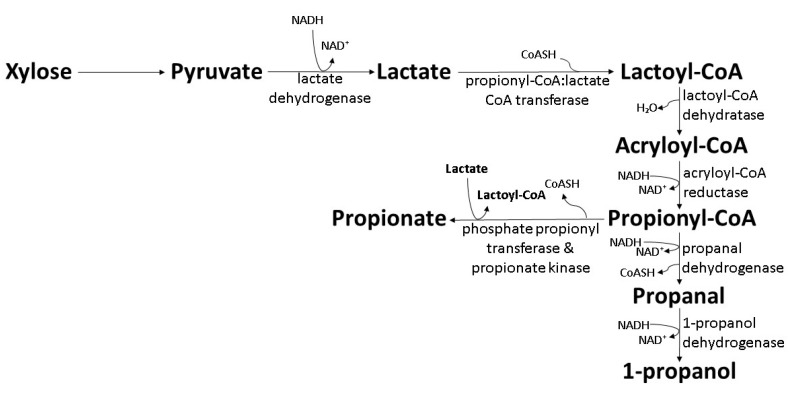
Biosynthesis of 1-propanol by wild-type *C. propionicumable* and *C. neopropionicumable* through the acrylate pathway [109,110].

*Clostridium propionicumable* and *Clostridium neopropionicumable* are able to use amino acids (alanine and serine), lactate, and ethanol as growth-promoting substances under anaerobic conditions [108,109]. *C. neopropionicumable* synthesizes a small amount of 1-propanol (0.06 g/L, 0.03 mol/mol) from ethanol with propionate and acetate as the main products [109]. By employing the bacterial mixture dominated by *Alkalibaculum bacchi* (34%) and *C. propionicumable* (54%), *C. propionicumable* produced 6.0 g/L 1-propanol and 1.0 g/L 1-butanol, whereas *A. bacchi* produced 8.0 g/L ethanol from syngas (the carbon source) and corn-steep liquor (the source of amino acids and minerals) [110]. The resulting 1-propanol was proposed to be the product from a two-step reduction in propionyl-CoA produced through the acrylate pathway by using the lactoyl-CoA that is not used for propionic acid synthesis (Figure 6) [109]. However, further experimentations are needed to provide essential information for a complete view of the biosynthesis pathway.

### 6.3. The Carboxylate Reduction Pathway in Clostridium

**Figure 7:**
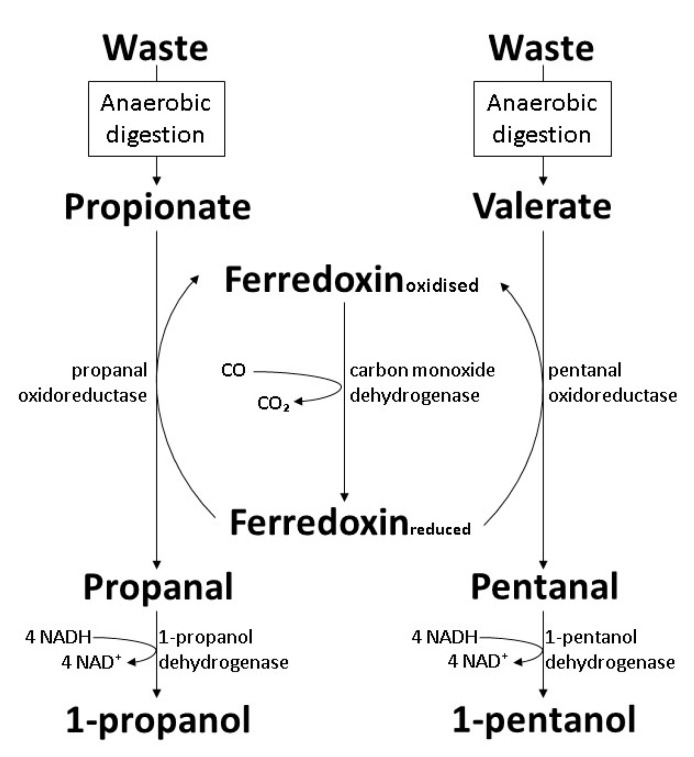
Biosynthesis of 1-propanol and 1-pentanol by wild-type *C. ragsdalei* through the carboxylate reduction pathway [112].

Anaerobic digestion by microbial consortia is a promising hydrogen production process where the members in the microbial community play different roles to convert raw materials into hydrogen under anaerobic conditions. As sterilization is commonly excluded from anaerobic digestion, organic acids produced by acetogens in the consortia cause decreased pH that disrupts the metabolic activity of hydrogen-producing bacteria [111]. *Clostridium ragsdalei* (ATCC BAA-622, DSM 15248) is an acetogen capable of synthesizing alcohols by ferredoxin-mediated carboxylate reduction. With the involvement of exogenous CO and ferredoxin, *n*-fatty acids up to six carbons in length can be reduced to corresponding alcohols (Figure 7). The concentration of produced 1-propanol reported was 1.7 g/L 1-propanol from propionic acid, with a conversion efficiency of 97%. However, the concentration of 1-pentanol obtained was merely 0.2 g/L, with a conversion efficiency of 82% [112].

## 7. Biosynthesis of 1-Propanol and 1-Pentanol by Genetic-Engineered *E. coli*

### 7.1. Co-Expression of the Citramalate and Threonine Pathway

**Figure 8:**
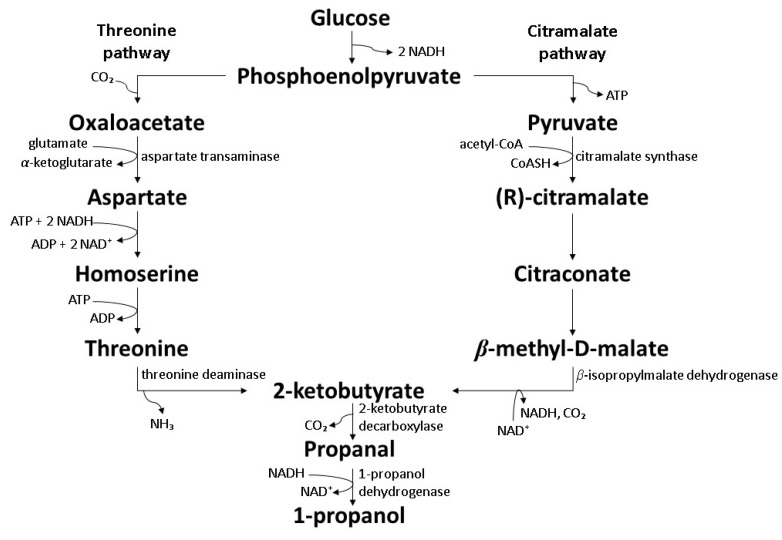
Biosynthesis of 1-propanol by genetically engineered *E. coli* BW25113 through co-expression of the citramalate and threonine pathway [115].

Numerous genetic engineering attempts were carried out for alkyl alcohols biosynthesis through the individual threonine or citramalate pathway and showed successful biosynthesis of 1-propanol from the intermediate 2-ketobutyrate in the pathways [113,114]. For greater industrial applicability, co-expression of both pathways was attempted in *E. coli* BW25113. The simultaneous operation of the pathways in a single host showed a synergic effect on 1-propanol production. The co-expression provided a larger 2-ketobutyrate pool for decarboxylation and reduction to 1-propanol (Figure 8). A high 1-propanol concentration of 8.0 g/L was reported with a 1-propanol yield of 0.15 g/g from glucose, which was higher than 0.09 and 0.11 g/g for individual threonine and citramalate pathway, respectively [115].

### 7.2. Interactive Elongation Cycle of 2-Ketoacids

**Figure 9:**
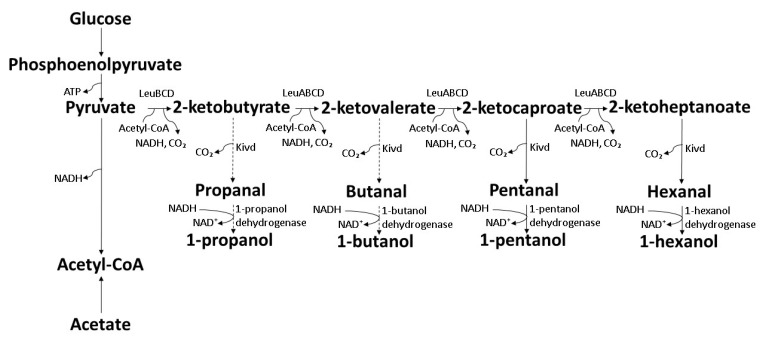
Biosynthesis of 1-pentanol by genetically engineered *E. coli* BW25113 through interactive elongation cycles of 2-letoacids [116]. Dash arrow indicates lower selectivity toward the reaction.

Biosynthesis of 1-pentanol was made possible by introducing *Lactococcus lactis* ketoisovalerate decarboxylase (Kivd) modified via saturated mutagenesis of the V461 key residue of the enzyme with glycine and serine into *E. coli* BW25113 to promote its selectivity toward 2-ketocaproate, which is the precursor for 1-pentanol. Besides lowered catalytic efficiency of the modified Kivd toward 2-ketoacids upstream of 2-ketocaproante, the increased supply of acetyl-CoA by acetate feeding encouraged 2-ketoacid elongation cycle for enhanced 1-pentanol production (Figure 9). The high specificity of this approach was implied by 90% 1-pentanol in the alcohol product mixture, equivalent to 2.2–2.4 g/L upon production harvest. The synthesis of alcohols with a longer alkyl chain was found to be minimized as further elongation of the 2-ketoacid was discouraged due to the active use of 2-kerocaproate for 1-pentanol synthesis [116].

### 7.3. Extended Dissimilation of Succinate

**Figure 10:**
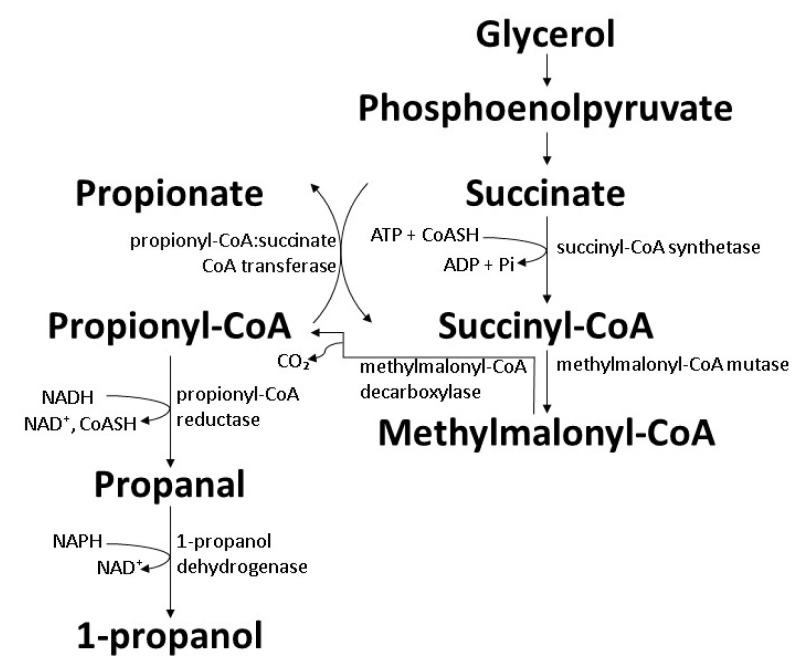
Biosynthesis of 1-propanol by genetically engineered *E. coli* BW25113 with activated SBM operon for extended dissimilation of succinate [118].

The sleeping beauty mutase (SBM) operon in *E. coli* is a four-gene operon (*sbm*-*ygfD*-*ygfG*-*ygfH*) that encodes various enzymes required in a cobalamin-dependent metabolic pathway for decarboxylation of succinate into propionate [117]. An activated chromosomal SBM operon encodes methylmalonyl-mutase (by *sbm*), methylmalonyl-CoA decarboxylase (by *ygfG*), and propionyl-CoA:succinate CoA transferase (by *ygfH*) in plasmid-free propanogenic *E. coli* BW25113 enabled extended dissimilation of succinate to synthesis 1-propanol (Figure 10). Glycerol favored solventogenesis over glucose due to the necessity of a solventogenic pathway as an auxiliary channel for redox balance upon glycerol dissimilation under anaerobic conditions. An anaerobic fed-batch strategy established by using the engineered *E. coli* strain produced high titers of 7.0 g/L 1-propanol, thus implying its high industrial applicability [118].

### 7.4. Acquired Carboxylate Reduction Pathway

**Figure 11:**
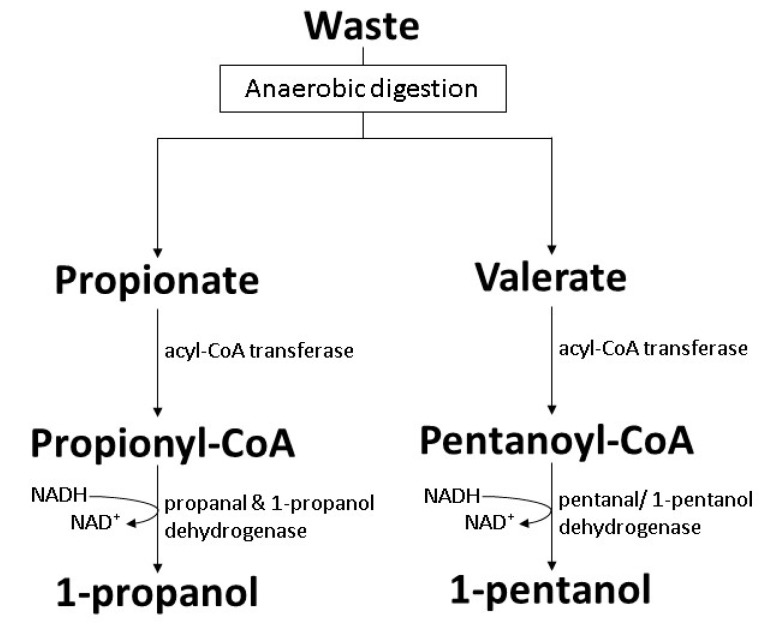
Biosynthesis of 1-pentanol by genetically engineered *E. coli* BL21(DE3 with acquired carboxylate reduction pathway [119].

Conversion of organic acids produced by acetogens during anaerobic digestion into other useful products is suggested to be beneficial as a solution to maintain the stability of the biogas production process. An *E. coli* BL21(DE3) strain harboring *Clostridium acetobutylicum* alcohol dehydrogenase (AdhE2) and *Megasphaera hexanoica* acyl-CoA transferase (ACT01_02765) was developed for conversion of the C_2_-C_8_ organic acids commonly found in anaerobic digestion into corresponding primary alcohols. The metabolic pathway is relatively simpler as it only involves two steps aided by two enzymes (Figure 11). Following the conversion rate of 1.1 for C_4_ acid into 1-butanol, the functional alcohol dehydrogenase and acyl-CoA transferase resulted in a promising conversion rate of 0.8 for both 1-propanol and 1-pentanol [119].

## 8. Alkyl Alcohol-Tolerant P(3HB-*co*-3HV)-Producing Bacteria

**Figure 12:**
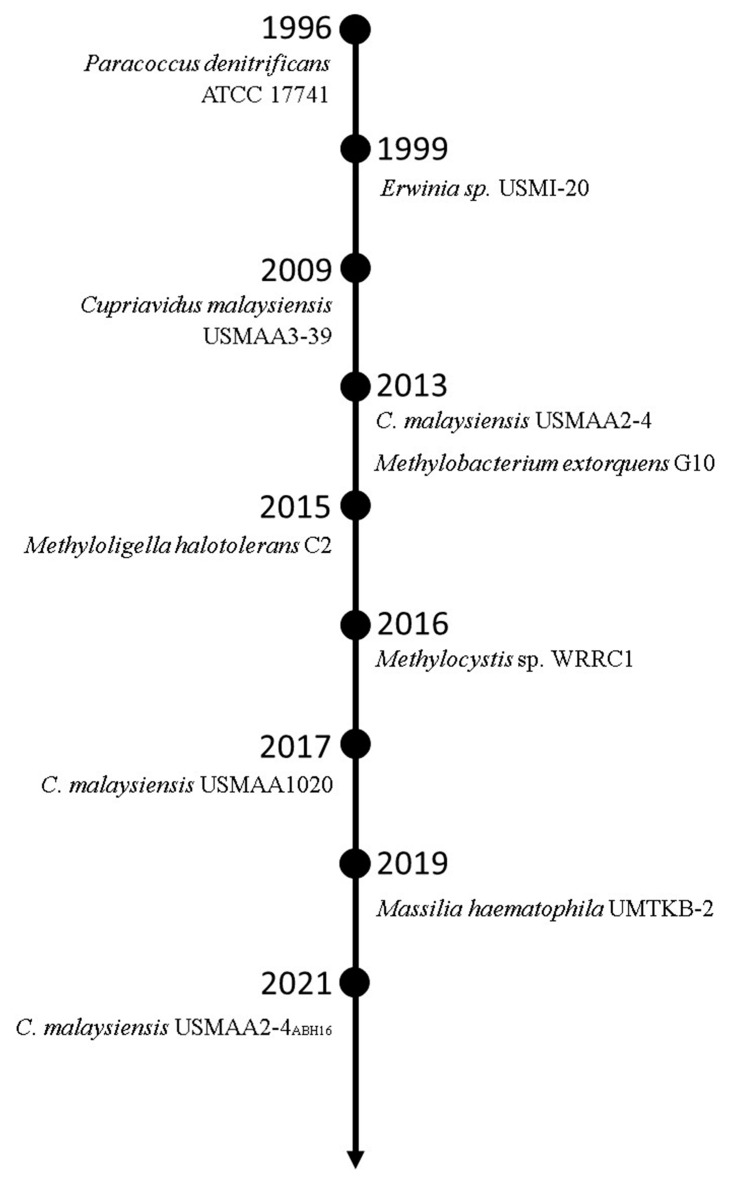
Timeline of the emergence of alkyl alcohol-tolerant P(3HB-*co*-3HV)-producing bacteria.

Alcohols are unsuitable to be employed as 3HV precursors for *C. necator* (also known as *Ralstonia eutropha*, *Alcaligenes eutrophus,* or *Wautersia eutropha*), which is the standard PHA-producing bacteria. Although *C. necator* H16 is capable of surviving methanol, ethanol, and propanol, extensive exposure to these alcohols is detrimental to PHA accumulation, thus resulting in lower biomass. The employment of 8.0 g/L 1-propanol, which is convertible into propionyl-CoA, contributed to merely 3 mol% 3HV with a 3HV yield of 0.14 g/g [13,120] (Table 2). The individual employment of 1-propanol and 1-pentanol also caused a remarkably high reduction in biomass and PHA content of *C. necator* DSM 545. The employment of 1-pentanol caused *C. necator* DSM 545 biomass and PHA content to decrease by 40% and 20%, respectively. Comparatively,1-propanol exerted a lower adverse effect compared to 1-pentanol, whereby its employment decreased *C. necator* DSM 545 PHA content by 10% with no negative influence on bacterial biomass [10] (Table 2). To overcome the limitation in 3HV precursor selection, isolation of alkyl alcohol-tolerant P(3HB-*co*-3HV)-producing bacteria is continuously attempted and has led to the discovery of various promising bacteria with the capability to use alkyl alcohols as 3HV precursors (Figure 12).

*P. denitrificans* ATCC 17741 was the first bacteria reported in 1996 for the use of alkyl alcohol as the 3HV precursor. *P. denitrificans* ATCC 17741 is a mixotrophic colorless sulfur bacterium capable of using 1-pentanol as the sole carbon source for growth and P(3HV) accumulation [14,121]. The study was conducted by maintaining the concentration of 1-pentanol at 1.6 g/L for 24 h. Approximately 6.8 g/L biomass with 1.2 g/L P(3HV) homopolymer was achieved [14] (Table 2).

*Erwinia* sp. USMI-20 was reported with its preference for alkyl alcohols instead of organic acids as 3HV precursors. *Erwinia* sp. USMI-20 achieved higher biomass with the co-employment of 1-propanol and 1-pentanol compared to that when palm oil was employed solely. A higher PHA content of 50 and 62 wt% was also achieved for 1-propanol and 1-pentanol, respectively, compared to 40 wt% and 34 wt% for propionic acid and valeric acid. 1-pentanol was more promising compared to 1-propanol as *Erwinia* sp. USMI-20 accumulated a higher 3HV fraction of 20 mol% from 1-pentanol compared to 6 mol% from 1-propanol. 1-pentanol can be employed as a substitute for valeric for *Erwinia* sp. USMI-20 owing to the higher 3HV yield of 0.43 g/g for 1-pentanol, which was 2-fold higher than that for valeric acid [60]. The production was scaled up to 10 L by employing 4.6 g/L palm oil and 1.4 g/L 1-pentanol, where 1-pentanol was added at 20 h post incubation. The 3HV fraction achieved was 20 mol% in 56 wt% PHA content of 5.4 g/L biomass, with 0.43 g/g 3HV yield [122] (Table 2).

Despite the negative influence observed for *C. necator*, there are several *Cupriavidus* sp. that are capable of using alkyl alcohols as 3HV precursors with no adverse effect on either bacterial biomass or PHA accumulation. *C. malaysiensis* USMAA2-4, *C. malaysiensis* USMAA1020, and *C. malaysiensis* USMAA9-39 are three PHA-producing bacteria favoring alkyl alcohols over organic acids for 3HV formation [123]. *C. malaysiensis* USMAA2-4 and *C. malaysiensis* USMAA1020 were able to accumulate 7–10 mol% 3HV from 1-pentanol [87,88,123,124,125] (Table 1). The 3HV yield of *C. malaysiensis* USMAA2-4 and its transformant strain harboring *C. necator* H16 *lipAB* genes was 0.22 g/g and 0.33 g/g, respectively, which were both higher than 0.14 g/g for *C. necator* H16 [13,87,88]. The sole employment of 1-pentanol resulted in a higher *C. malaysiensis* USMAA9-39 PHA content of 46 wt% compared to 37 wt% for valeric acid. Despite the 1-fold lower *C. malaysiensis* USMAA9-39 biomass resulting from the co-employment of 1-pentanol with oleic acid, the 3HV yield of 0.44 g/g from 1-pentanol was comparable to 0.42 g/g from valeric acid and a high 3HV composition of 24 mol% was achieved [76] (Table 2).

*M. extorquens* G10 demonstrated the production of P(3HB-*co*-3HV) from an alkyl alcohol mixture of C1 and C5 alcohol. A 4 L production of P(3HB-*co*-3HV) from a methanol-pentanol mixture by *M. extorquens* G10 showed a promisingly high PHA concentration of 7.5–18.0 g/L. The carbon mixture was supplemented fractionally based on the dissolved oxygen peaks observed. With an increased portion of 1-pentanol from 2 to 20 mol%, the biomass decreased with association to reduction in PHA content from 40.0 to 25.0 g/L and 45 to 30 wt%, respectively. Despite the negative influence on biomass and PHA content, 3HV composition of 14–50 mol% was achieved [90] (Table 2).

*M. halotolerans* C2 demonstrated P(3HB-*co*-3HV) production from C1, C2, and C5 alkyl alcohol. P(3HB-*co*-3HV) production by *M. halotolerans* C2 through fractional feeding of methanol-ethanol mixture resulted in increased 3HV composition from 2 to 51 mol% parallel to increased 1-pentanol supply from 5 to 15 % v/v methanol. A considerably high PHA content of 73–98 wt% was accumulated by the bacterium [91] (Table 2).

P(3HB-*co*-3HV) production by *Methylocystis* sp. WRRC1 from methane and 1-pentanol demonstrated a 0.17 g/g 3HV yield from 1.0 g/L 1-pentanol. The 6-fold lower consumption of methane by the bacteria with the co-employment of 1-pentanol compared to that of sole employment of methane denoted the preference of the bacteria for 1-pentanol over methane. However, 1-pentanol is non-competitive against valerate where *Methylocystis* sp. WRRC1 achieved a 1-fold higher 3HV concentration with the co-employment of sodium valerate compared to that of 1-pentanol. On the other hand, the co-employment of sodium valerate did not cause reduced methane consumption and contributed to a higher 3HB concentration [86] (Table 2).

*M. haematophila* (also known as *Naxibacter haematophila*) UMTKB-2, a slow-growing bacterium, was also reported with the capability to use 1-pentanol for 3HV accumulation with a preference for 1-pentanol over valeric acid and sodium valerate. The co-employment of 1-pentanol resulted in 2-fold and 11-fold higher biomass and PHA content compared to that of valeric acid and sodium valerate, respectively. Upon optimization by using response surface methodology, *M. haematophila* UMTKB-2 achieved 7 mol% 3HV with 0.40 g/g 3HV yield. Unlike the PHA accumulation process of *Cupriavidus* sp. that ends within 48–72 h, 122 h was needed for optimum P(3HB-*co*-3HV) accumulation by *M. haematophila* UMTKB-2 [89] (Table 2).

## 9. Mode of Action of 1-Propanol and 1-Pentanol on Proteins

Short-chain alcohols exert a hydrophobic effect by interacting with proteins and lead to the structural unfolding of the protein [126]. Changes in membrane fluidity ensue due to the direct insertion of lipophilic agents into the cellular membrane after direct physicochemical interaction with alcohols. This induces adaptive membrane alteration by changing the fatty acid composition of the membrane [127]. Impaired inner membrane integrity associated with depletion in proton motive force due to the increased proton motive force demand for chemical, osmotic and mechanical adjustment induces the *psp* operon to prevent proton loss. As a result, the cells experience a metabolic shift to anaerobic respiration together with downregulation of motility for adjustment and maintenance of energy as well as for proton motive force usage [128]. The extent of water exclusion is greater with increasing alkyl groups of the alcohol, which is non-polar. By considering the hydrophobic effect of methanol < ethanol < propanol = butanol, pentanol may exert a similar hydrophobic effect on protein and result in pentanol-induced protein unfolding [126]. Furthermore, pentanol is capable of inactivating membrane proteins such as transporters but rarely causes structural changes to the cell membrane [129].

## 10. Mechanisms Involved in Alcohols Tolerance

Aliphatic alcohols, aromatic compounds, or other organic solvents are toxic to bacteria when present in high concentrations. Nevertheless, certain bacteria are able to thrive in the high concentration of such toxic organic chemicals. Bacterial solvent tolerance is a multifactorial process that involves gene expression and subsequent physiological changes to respond to stress conditions. Extrusion of the toxic compounds from the cell to the external environment and reduced cell membrane permeability to prevent further influx of toxic compounds are the relevant mechanisms to survive alcohol stress.

### 10.1. Changes in the Cell Membrane

Alcohol-induced cell leakage of magnesium and nucleotides is the primary damaging action that affects bacterial viability in alcohols [130]. As alcohols interact with the cell membrane and decrease the degree of membrane organization, proteins that participate in membrane structure organization and surface stabilization are critical in alcohol tolerance [131]. Isomerase incorporates fatty acids into the phospholipid headgroups of the phospholipid bilayer and causes isomerization of *cis* unsaturated fatty acids to *trans* unsaturated fatty acids to form a denser membrane, as demonstrated by *Pseudomonas* and *Vibiro* [132]. Changes in cell membrane composition that attributed to increased *cis*-11 vaccenic acid (18:1) or *cis*-9 oleic acid (18:1) with a corresponding decrease in palmitic acid (16:0) were demonstrated for *E. coli*, *Lactobacillus homohiochii,* and *Saccharomyces cerevisiae* [131,133,134]. The synthesis of phosphatidylethanolamine by *Zymomonas mobilis* was partially inhibited in the presence of alcohols. As a result, a membrane with an elevated proportion of acidic phospholipids (phosphatidylglycerol and cardiolipin) and an overall reduction in the phospholipid:protein ratio is synthesized, thus increasing the efficiency of efflux pumps in alcohol extrusion [135,136].

### 10.2. Stress Response System

Exposure to alkyl alcohols leads to changes in the level of expression of certain genes as responses to stress for adaptation. As demonstrated in *E. coli*, exposure to 1-butanol causes downregulation of several genes related to histidine, leucine, arginine, tryptophan, and methionine biosynthesis and transport, thus leading to a significantly lower level of related proteins. Downregulation of genes related to amino acids metabolism is an indicator for bacterial growth inhibition in alcohols. As opposed to that, *opp* operon (*oppABCDF*) that encodes the components in a polyamine-induced oligopeptide ABC transport system is upregulated for the transport of hydrophilic substances to compensate for the hydrophobic pressure exerted by alcohols [137,138]. Genes responsible for response to heat shock and extracytoplasmic stress (*cpx* regulon) are upregulated, and periplasmic chaperone Spy is encoded to respond to protein misfolding activity [139,140,141,142]. Increased isobutanol tolerance of *C. acetobutylicum* is also conferred to overexpression of genes related to heat shock [139,143]. Genes related to the membrane and periplasmic space carbohydrate transport and metabolisms are upregulated to transport and phosphorylate hexoses and release the phosphate esters into the cytoplasm, probably as a repair mechanism for damaged bilayer [139,144]. Furthermore, the upregulation of genes from the 13-member *nuo* operon and 5-member *cyo* operon is also an indicator for the increased requirement of energy or disruption of respiratory efficiency upon exposure to 1-butanol [139]. However, the operons are downregulated when exposed to isobutanol [137]. Exposure to ethanol causes induced expression of *psp* operon to restore proton motive force lost due to disruption of the cell membrane by ethanol, but the expression level remains unchanged for isobutanol [128,137].

## 11. Challenges in Wide Implementation of Alkyl Alcohols as 3HV Precursors

Low alcohol tolerance due to alcohol toxicity is the major drawback for the employment of alkyl alcohols as 3HV precursors. Isolation of novel PHA-producing bacteria with substantial alcohol tolerance is a continuous effort in developing production processes with higher economic feasibility. With established primary alkyl alcohol bioproduction processes, the employment of alkyl alcohols also contributes to sustainability. Alcohol tolerance involves complex regulatory systems, and knowledge from cell-wide stress response is still in demand. Theoretically, genetic engineering can be adopted to create an alkyl alcohol-tolerant PHA-producing bacteria by either introducing *pha* genes into an alkyl alcohol-tolerant host or modulating alcohol tolerance of a non-alkyl alcohol-tolerant PHA-producing bacteria. Comparatively, the former approach is more rational as alcohol tolerance involves complex systems and is not economically feasible for commercial importance.

Although genetic-engineered *E. coli* with mutated *rpoA* gene was constructed successfully to produce products with commercial importance such as 1-butanol, the attempt was based on extensive studies on the *rpoA* gene and its roles in phenotypic changes of *E. coli* [145,146,147,148,149]. Owing to numerous studies on the incorporation of *pha* genes into *E. coli,* which demonstrated successful production of various PHA, such approaches can be adopted for the construction of alkyl alcohol-tolerant strains with acquired PHA-producing ability [150,151,152]. However, a candidate strain with broad substrate preference is preferred for production process establishment with different substrates and fermentation strategies. The capability to use wastes with high carbon content will be an added value for higher industrial applicability owing to its sustainability and higher economic feasibility compared to pure carbon sources [87].

Despite the promising potential shown by the known alkyl alcohol-tolerant P(3HB-*co*-3HV)-producing bacteria, scaling up the production remains challenging. As low 3HV compositions are commonly reported for shake flask scale production, various production strategies have to be adopted to increase the molar fraction of 3HV. Fed-batch production strategies that enable the addition of alkyl alcohols eventually are practically preferred to achieve high 3HV composition of P(3HB-*co*-3HV) and at the same time minimize the negative influences caused by the relative toxicity of alkyl alcohol. However, some of the bacteria that depicted decreased biomass and PHA content with the employment of alkyl alcohol at low concentration or with a preference for organic acid sodium salt over alkyl alcohol have low applicability as candidate P(3HB-*co*-3HV) producers when alkyl alcohols are to be employed. In addition, more studies on large-scale P(3HB-*co*-3HV) production involving alkyl alcohols are still in demand to compare their industrial practicality as alternative 3HV precursors for organic acids in terms of sustainability and economic feasibility.

## 12. Concluding Remark

The high sale price of P(3HB-*co*-3HV) has been the major obstacle to commercialization. Although various carbon sources have been explored, limited precursor choice due to the domination by propionate and valerate has caused the development of diverse P(3HB-*co*-3HV) production to reach a bottleneck. With increasing studies reporting the discovery of alkyl alcohol-utilizing PHA-producing bacteria with promising bioconversion efficiency of 1-propanol and 1-pentanol into 3HV, the toxicity of alkyl alcohols and low 3HV yield are no longer the major concern. Future attempts should focus on continuous searching of alkyl alcohols tolerant PHA-producing bacteria to discover more promising wild-type strains. Moreover, genetic engineering of bacterial metabolic pathways to achieve successful or higher bioconversion rate of alkyl alcohols into 3HV is also important to overcome low bacterial viability and alcohol-3HV bioconversion efficiency. However, more studies are required for techno-economic assessment to compare to what extent 1-propanol and 1-pentanol could contribute to higher economic feasibility than propionate and valerate.

## Figures and Tables

**Table 1:** Properties improvement after the incorporation of a secondary (and tertiary) component into P(3HB-*co*-3HV) and their potential applications.

Incorporated Components ^A^	Changes in the Properties	Potential Applications	Ref.
***α*-P(3HB)** Incorporation method: Solvent casting 3HV fraction: 10 mol%	P(3HB-*co*-3HV):*α*-P(3HB) (100:0 → 50:50) Melting temperature: 145 → 133 °C Degree of crystallinity: 61% → 30% Tensile strength: 27 → 7 MPa Elongation at break: 1% → 29% Young’s modulus: 1500 → 240 MPa Enzymatic degradation: 85% → 94%	Packaging material	[25]
**AS** Incorporation method: Solvent casting 3HV fraction: 59 mol%	P(3HB-*co*-3HV)/P(3HB-*co*-3HV):AS Melting temperature: 275.84 °C/294.97 °C Degree of crystallinity: 98.96%/98.23% Free radical scavenging activity (24 h): 1%/14% Incubation biodegradation (day 6): smooth surface/small pits	Therapeutic implant	[26]
**CNC** Incorporation method: Solvent casting 3HV fraction: 12 mol%	P(3HB-*co*-3HV):CNC (100:0 → 94:6) Melting temperature: 136.8 → 151.1 °C Crystallization temperature: 96.5 → 101.2 °C Degree of crystallinity: 49.9% → 57.5% Water vapor transmission rate: 308 → 115 g m^−2^ day^−1^ Oxygen transfer rate: 425 → 113 cm m^−2^ day^−1^	Packaging material	[27]
**DDGS or Misc** Incorporation method: Twin screw extrusion 3HV fraction: 5 mol%	P(3HB-*co*-3HV):DDGS (100:0/85:15/75:25) Tensile strength: 8.5 MPa/6.0 MPa/4.8 MPa Young’s modulus: 3.9 GPa/3.9 GPa/3.8 GPa Flexural strength: 7.0 MPa/5.8 MPa/4.7 MPa Flexural modulus: 4.8 GPa/4.6 GPa/4.4 GPa CO_2_ evolution (day 320): 155 mg/175 mg/200 mg Marine biodegradation (day 320): 73%/90%/100% P(3HB-*co*-3HV):Misc (85:15/75:25) Tensile strength: 8.8 MPa/8.9 MPa Young’s modulus: 5.9 GPa/7.7 GPa Flexural strength: 7.8 MPa/7.4 MPa Flexural modulus:5.6 GPa/6.6 GPa CO_2_ evolution (day 320):175 mg/180 mg Marine biodegradation (day 320): 84%/88%	Packaging material	[28]
**Eugenol** Incorporation method: Electrospinning 3HV fraction: 3 mol%	P(3HB-*co*-3HV):Eugenol (100:0 → 85:15) Temperature of 5% weight loss: 276.6 → 160.8 °C Degradation temperature: 304.7 → 293.3 °C Mass loss at degradation temperature: 61.01% → 76.36% Water vapor permeability: 4.05 × 10^14^→ 0.95 × 10^14^ Kg m m^−2^ s^−1^ Pa^−1^ Limonene vapor permeability: 3.75 → 0.81 Kg m m^−2^ s^−1^ Pa^−1^ Water vapor permeance: 5.87 → 1.33 Kg m m^−2^ s^−1^ Pa^−1^ Limonene vapor permeance: 5.44 → 1.14 Kg m m^−2^ s^−1^ Pa^−1^ Tensile strength: 1252 → 1897 MPa Elongation at break: 2.0% → 2.5% Young’s modulus: 18.1 → 26.5 MPa *S. aureus* growth: 5.16 → 3.45 log(CFU mL^−1^) *Escherichia coli* growth: 5.79 → 3.88 log(CFU mL^−1^)	Antimicrobial food packaging	[29]
**HA** Incorporation method: Melt-pressing 3HV fraction: 8–24 mol%	P(3HB-*co*-3HV), 0→24 mol% 3HV Melting temperature: 170 → 129 °C Degree of crystallinity: 69% → 55% P(3HB-*co*-3HV):HA (30:70), 0 → 24 mol% 3HV Tensile strength: 67 → 23 MPa Elongation at break: 2.65% → 3.84% Young’s modulus: 2.52 → 0.47 GPa	Bone implant	[30]
**MAT** Incorporation method: Solvent casting 3HV fraction: 4 mol%	P(3HB-*co*-3HV):MAT (100:0 → 95:5) Melting temperature: 168.58 → 130.91 °C Glass transition temperature: −2.03 → −6.61 °C Crystallization temperature: 46.15 → 46.98 °C Degree of crystallinity: 53.7% → 36.8%	Packaging material	[31]
**MCPA** Incorporation method: Melt-blending and hot-pressing 3HV fraction: 3 mol%	P(3HB-*co*-3HV)-MCPA (95:5/90:10/85:15) Melting temperature 1: 123.2 °C/124.1 °C /NA Melting temperature 2: 150.7 °C/150.7 °C/140.9 °C Enthalpy of fusion 1:1944 J g^−1^/2482 J g^−1^/NA Enthalpy of fusion 2:1745 J g^−1^/1745 J g^−1^/1509 J g^−1^ Glass transition temperature 1: −28.2 °C/−28.0 °C/−27.4 °C Glass transition temperature 2: 48.6 °C/47.9 °C/36.9 °C Crystallization temperature: 102.4 °C/102.2 °C/99.0 °C Chlorine loss: 0.3%/1.3%/1.7% MCPA loss: 5.1%/7.4%/9.7% P(3HB-*co*-3HV) loss before bond scission: 20.6%/29.7%/38.8% P(3HB-*co*-3HV) loss after bond scission: 2.8%/2.4%/2.4%	Mulch	[32]
mPEG Incorporation method: Transesterification 3HV fraction: 12 and 33 mol%	P(3HB-*co*-3HV):mPEG, 12 mol%/33 mol% 3HV Number average molecular weight: 8980/4980 Weight average molecular weight: 6200/2650 Polydispersity index: 1.44/1.84 Melting temperature of P(3HB-*co*-3HV) block: 140.5 °C/133.6 °C Melting temperature of mPEG block: 49.1 °C/49.3 °C Particle size: 162 nm/125 nm Encapsulation efficiency: 43%/57% Cytotoxicity (100 → 500 µg/mL nanoparticles): 94% → 80%/88% → 78%	Drug delivery carrier	[24]
**NH_2_-*g*-collagen or** **PHEMA-*g*-collagen** Incorporation method: Solvent casting followed by solute leaching technique 3HV fraction: 12 mol%	Porous P(3HB-*co*-3HV) Decomposition temperature at 10% weight loss: 263.15 °C Collagen concentration: NA Ag/BSA load: 0.037 µg cm^−2^ Surface roughness: 0.1983 µm P(3HB-*co*-3HV)--*g*-PHEMA-*g*-collagen Decomposition temperature at 10% weight loss: 264.60 °C Collagen concentration: 29.93 µg cm^−2^ Ag/BSA load: 0.29 µg cm^−2^ Surface roughness: NA P(3HB-*co*-3HV)-*g*-NH_2_-*g*-collagen Decomposition temperature at 10% weight loss: 256.15 °C Collagen concentration: 55.16 µg cm^−2^ Ag/BSA load: 0.26 µg cm^−2^ Surface roughness: 0.2643 µm	Bone implant	[33]
**NR** Incorporation method: Twin screw extrusion 3HV fraction: 3 mol%	P(3HB-*co*-3HV):NR (100:0/85:15) Melting temperature: 172.05 °C/171.95 °C P(3HB-*co*-3HV) glass transition temperature: 5.65 °C/6.05 °C NR glass transition temperature: NA/−64.5 °C Crystallization temperature: 120.85 °C/119.45 °C Degree of crystallinity: 74.7%/61.6% Tensile strength: 43 MPa/26 MPa Elongation at break: 8%/16% Notched impact strength: 15 J m^−1^/14 J m^−1^ Secant modulus: 12 GPa/0.9 GPa	Packaging material	[34]
**PBAT** Incorporation method: Conventional injection molding or microcellular injection molding 3HV fraction: NA	Solid P(3HB-*co*-3HV):PBAT (98.5:1.5 → 30:70) Melting temperature: 166.2 → 170.4 °C Cold crystallization temperature: NA → 44.7 °C Degree of crystallinity: 78% → 29% Specific toughness: 5.3 x 10^−4^ → 7.1 x 10^−2^ MPa kg^−1^ m^−3^ Elongation at break: 2.7% → 555.7% Specific tensile strength: 3.2 × 10^−2^ → 1.5 x 10^−2^ MPa kg^−1^ m^−3^ Specific Young’s modulus: 2.2 → 0.5 MPa kg^−1^ m^−3^ Microcellular P(3HB-*co*-3HV):PBAT (98.5:1.5 → 30:70) Melting temperature: 167.1 → 169.6 °C Cold crystallization temperature: NA → 45.7 °C Degree of crystallinity: 80% → 25% Specific toughness: 3.8 x 10^−4^ → 5.8 x 10^−2^ MPa kg^−1^ m^−3^ Elongation at break: 2.2% → 493.9% Specific tensile strength: 2.7 × 10^−2^ → 1.3 x 10^−2^ MPa kg^−1^ m^−3^ Specific Young’s modulus: 2.1 → 0.5 MPa kg^−1^ m^−3^	Packaging material	[35]
**PBS** Incorporation method: Solvent casting 3HV fraction: 14 mol%	P(3HB-*co*-3HV):PBS (100:0 → 40:60) Crystallization time at 60 °C: 8 → 14.5 min Overall crystallization constant: 3.13 × 10^−2^ → 2.22 × 10^−3^ min^−n^ Avrami index: 2.57 → 2.67	Packaging material	[36]
**PBS-DCP** Incorporation method: Compression molding 3HV fraction: 13 mol%	P(3HB-*co*-3HV):PBS (100:0 → 70:30) Tensile strength: 22 → 23 MPa Elongation at break: 4.5% → 6.5% 80 wt%P(3HB-*co*-3HV)–20 wt%PBS:DCP (100:0 → 99:1) Tensile strength: 25 → 27 MPa Elongation at break: 8% → 350% Notched Izod impact toughness: 2.8 → 5.5 kJ m^−2^ Flexural strength: 39 → 30 MPa Flexural modulus: 1.2 → 0.6 GPa	Packaging material	[37]
**PCL** Incorporation method: Solvent casting 3HV fraction: 7 mol%	P(3HB-*co*-3HV)/PCL Number average molecular weight: 127,000/56,400 Weight average molecular weight: 470,000/163,300 Melting temperature: 151.2 °C/64.0 °C Glass transition temperature: 5.2 °C/−61.0 °C Crystallization temperature: 97.0 °C/22.2 °C P(3HB-*co*-3HV):PCL (100:0 → 50:50) Isothermal crystallization temperature: 120 → 120 °C Overall crystallization constant: 2.20 × 10^−7^ → 1.00 × 10^−8^ s^−n^ Avrami index: 2.80 → 2.66	Packaging material	[38]
**PDLLA-PEG** Incorporation method: Compression molding 3HV fraction: 1 mol%	P(3HB-*co*-3HV):PDLLA (100:0 → 30:70) Melting temperature: 157.8 → 169.8 °C Degree of crystallinity: 53.6 → 9.9 °C Tensile strength: 19.7 → 49.7 MPa Elongation at break: 0.17% → 2.07% Flexural strength: 39.1 → 75.0 MPa Flexural modulus:3646 → 3507 MPa Burial biodegradation (day 30): 0% → 1% 30 wt%P(3HB-*co*-3HV)–70 wt%PDLLA:PEG (90:10 → 80:20) Melting temperature: 171.2 → 170.8 °C Degree of crystallinity: 10.5 → 13.0 °C Tensile strength: 29.7 → 24.1 MPa Elongation at break: 28.7% → 237.0% Flexural strength: 36.1 → 5.48 MPa Flexural modulus: 1127 → 220 MPa Burial biodegradation (day 30): 3% → 11%	Biomedical, agricultural and packaging material	[39]
**PEG** Incorporation method: Solvent casting 3HV fraction: 4 mol%	P(3HB-*co*-3HV):PEG (100:0 → 20:80) Melting temperature: 163.2 → 145.0 °C Enthalpy of fusion: 89.62 → 1.63 J g^−1^	Drug delivery carrier	[22]
**PEG** Incorporation method: Solvent casting 3HV fraction: NA	P(3HB-*co*-3HV) Melting temperature: 90 °C Initial thermal degradation temperature: 220 °C Final thermal degradation temperature: 255 °C Tensile strength: 10.3 MPa Elongation at break: 13.3% Cytotoxicity: 20% P(3HB-*co*-3HV):PEG (4:1) Cytotoxicity: 0%–10%	Skin grafting	[40]
**PLA-CNT** Incorporation method: High-speed spinning 3HV fraction: 2 mol%	P(3HB-*co*-3HV)/PLA Melting temperature: 172 °C/170 °C Glass transition temperature: 5 °C/64 °C Enthalpy of fusion: 92.8 J g^−1^/44.2 J g^−1^ Crystallization temperature: 122 °C/112 °C Decomposition temperature: 303 °C/382 °C Izod impact strength: 1.99 kJ m^−2^/2.14 kJ m^−2^ Flexural strength: 47.70 MPa/58.07 MPa Flexural modulus: 3.48 GPa/2.94 GPa 80 wt%P(3HB-*co*-3HV)–20 wt%PLA:CNT (100:0 → 99:1) Melting temperature: 169 → 168 °C Glass transition temperature: −2 → −2 °C Enthalpy of fusion: 44.11 → 48.10 J g^−1^ Crystallization temperature: 112 → 122 °C Decomposition temperature: 379 → 380 °C Izod impact strength: 4.10 → 2.46 kJ m^−2^ Flexural strength: 51.60 → 61.01 MPa Flexural modulus: 3.10 → 3.25 GPa Electrical conductivity: 8.67 × 10^−14^ → 2.79 × 10^−2^ S m^−1^ Reflectivity (frequency): 0 dB (NA) → −15 dB (11 GHz)	Electrical and electromagnetic	[41]
**PLA-nanoclay** Incorporation method: Twin screw extrusion 3HV fraction: NA	P(3HB-*co*-3HV):PLA (15:85 → 30:70) Melting temperature: 154.75 → 156.40 °C Cold crystallization temperature: 133.45 → 121.89 °C Degree of crystallinity: 1.98% → 4.33% Tensile strength: 52.5 → 47.5 MPa Elongation at break: 9.0% → 6.0% Young’s modulus: 1700 → 1750 MPa P(3HB-*co*-3HV)-PLA:nanoclay (15:85 → 30:70) Melting temperature: 156.52 → 157.43 °C Cold crystallization temperature: 129.09 → 111.04 °C Degree of crystallinity: 13.05% → 18.40% Tensile strength: 49.2 → 48.0 MPa Elongation at break: 8.5% → 4.0% Young’s modulus: 2060 → 2060 MPa	Packaging material	[42]
**PPC** Incorporation method: Solvent casting 3HV fraction: 5 mol%	P(3HB-*co*-3HV):PPC (100:0 → 20:80) Melting temperature: 163 → 162 °C Thermal decomposition temperature: 199 → 190 °C Maximum mass loss rate temperature: 286 → 267 °C Burial biodegradation: 100% (day 12) → 85% (day 30)	Packaging material	[43]
**starch, cellulose or alginate** Incorporation method: Solvent casting 3HV fraction: 6 mol%	P(3HB-*co*-3HV)-starch (100:0 → 30:70) Tensile strength: 25 → 1 MPa Elongation at break: 8% → 4% Young’s modulus: 181 → 4 MPa Density: 0.974 → 1.243 g cm^−3^ Solubility: 0% → 6.0% Water absorption capacity: 0% → 21.0% Burial biodegradation (day 30): 10% → 100% Immersion biodegradation (day 30): 23% → 100% P(3HB-*co*-3HV)-cellulose (100:0 → 30:70) Tensile strength: 25 → 1 MPa Elongation at break: 8% → 3% Young’s modulus: 181 → 7 MPa Density: 0.974 → 1.212 g cm^−3^ Solubility: 0% → 1.7% Water absorption capacity: 0% → 4.7% Burial biodegradation (day 30): 10% → 70% Immersion biodegradation (day 30): 23% → 100% P(3HB-*co*-3HV)-arginate (100:0 → 30:70) Tensile strength: 25 → 1 MPa Elongation at break: 8% → 2% Young’s modulus: 181 → 3 MPa Density: 0.974 → 1.053 g cm^−3^ Solubility: 0% → 19.0% Water absorption capacity: 0% → 33.0% Burial biodegradation (day 30): 10% → 80% Immersion biodegradation (day 30): 21% → 100%	Mulch	[44]
**ZnO** Incorporation method: Melt-mixed compression molding, electrospinning or coating 3HV fraction: 3 and 18 mol%	P(3HB-*co*-3 mol%3HV)/ P(3HB-*co*-18 mol%3HV) Melting temperature: 168.7 °C/170.9 °C Decomposition temperature: 290.8 °C/283.1 °C Crystallization temperature: 114.7 °C/101.0 °C Degree of crystallinity: 66%/63% Tensile strength: 33.9 MPa/18.5 MPa Elongation at break: 1.5%/1.3% Young’s modulus: 2.6 GPa/2.2 GPa L*, a*, b*: 82.3, 1.4, 17.7/32.7, 6.7, 10.2 PHBVs-D/PHBVs-P/PHBVs-C ^B^ Melting temperature: 166.9 °C/166.5 °C/169.0 °C Decomposition temperature: 271.3 °C/270.3 °C/270.8 °C Crystallization temperature: 112.1 °C/111.6 °C/118.0 °C Degree of crystallinity: 50%/51%/35% Tensile strength: 12.5 MPa/34.8 MPa/22.6 MPa Elongation at break: 6.5%/2.3%/6.2% Young’s modulus: 1.5 GPa/2.1 GPa/1.4 GPa L*, a*, b*: 56.9, 9.0, 25.3/58.4, 8.5, 25.1/72.5, 3.8, 24.7	Active food packaging and food contact surface applications	[45]
ZnO Incorporation method: Laser 3D molding 3HV fraction: NA	P(3HB-*co*-3HV)-ZnO (100:0→95:5) Melting temperature: 171 → 158 °C Decomposition temperature: 261.2 → 288.7 °C Strain:14.0% → 9.5% Stress: 3.5 → 4.5 MPa Compression strength: 4 → 5 MPa Compression modulus: 60 → 80 MPa Bacterial inhibition rate (day 5): 2.5% → 79.0% Zn^2+^ release in deionized water (day 7): 0.19 → 0.34 mg L^−1^	Bone repair	[46]

^A^ Synthetic atactic poly(3-hydroxybutyrate) (*α*-P(3HB)), bovine serum albumin capped silver (Ag/BSA), ascorbic acid (AS), cellulose nanocrystals (CNC), carbon nanotubes (CNT), dicumyl peroxide (DCP), distillers’ dried grains with solubles (DDGS), hydroxyapatite (HA), organophilic attapulgite (MAT), *Miscanthus* (Misc), 2-methyl-4-chlorophenoxyacetic acid (MCPA), monomethoxy poly(ethylene glycol) (mPEG), natural rubber (NR), poly(*ε*-caprolactone) (PCL), poly(d,l-lactide) (PDLLA), poly(butylene succinate) (PBS), poly(butylene adipate-*co*-terephthalate) (PBAT), poly(ethylene glycol) (PEG), poly(2-hydroxyl ethyl methacrylate) (PHEMA), poly(lactic acid) (PLA), poly(propylene carbonate) (PPC), not available (NA). ^B^ Melt-mixed compress molded P(3HB-*co*-3 mol%3HV):P(3HB-*co*-18 mol%3HV):ZnO (70:24:6) (PHBVs-D), electrospun P(3HB-*co*-18 mol%3HV):ZnO (50:50) (PHBVs-P), P(3HB-*co*-18 mol%3HV):ZnO (50:50) coating on compressed molded P(3HB-*co*-3 mol%3HV) (PHBVs-C).

**Table 2:** P(3HB-co-3HV) production by bacteria from various 3HV precursors.

Microorganisms and Carbon Sources	Biomass (g/L)	PHA Content		3HV Composition		3HV Yield (g/g)	Ref.
		(wt%)	(g/L)	(mol%)	(g/L)		
Organic acids							
*Bacillus aryabhattai* PHB10 Glucose (20.0 g/L) Propionic acid (0.7 g/L)	3.9	72	2.8	-	-	-	[28]
*Bacillus thuringiensis* R-510 Glucose (23.5 g/L) Propionic acid (1.0 g/L)	2.9	21	0.6	41	0.2	0.25	[69]
*C. necator* DSM 545 Waste glycerol (20.0 g/L) Propionic acid (4.0 g/L)	4.5	57	2.6	25	0.7	0.16	[70]
*C. necator* DSM 545 Butyric acid (246.0 g/L) Propionic acid (186.0 g/L)	65.9	88	58	36	20.8	0.11	[71]
*C. necator* NRRL B 14690 Fructose (40.0 g/L) Propionic acid (4.0 g/L)	8.2	73	6.0	23	1.4	0.35	[72]
*C. necator* NCIMB 11599 Glucose (maintained at 10.0–20.0 g/L) Propionic acid (0.52 mol/mol glucose)	112.3	57	64.0	14	15.7	-	[73]
*Erwinia* sp. USMI-20 Palm oil (4.6 g/L) Propionic acid (1.9 g/L)	4.2	40	1.7	34	0.6	0.30	[60]
Activated sludge mixed culture Acetic acid, lactic acid, propionic acid	-	-	-	31–66	-	-	[74]
*Bacillus cereus* RCL 02 Glucose (25.0 g/L) Valeric acid (1.9 g/L)	8.1	72	5.8	15	0.9	0.46	[75]
*C. malaysiensis* USMAA9-39 Oleic acid (6.5 g/L) Valeric acid (0.9 g/L)	5.2	43	2.2	17	0.4	0.42	[76]
*C. necator* DSM 545 Waste glycerol (20.0 g/L) Valeric acid (4.0 g/L)	5.3	64	3.4	31	1.1	0.26	[70]
*C. necator* NRRL B 14690 Fructose (40.0 g/L) Valeric acid (4.0 g/L)	7.2	40	2.9	62	1.8	0.45	[72]
*Erwinia* sp. USMI-20 Palm oil (4.6 g/L) Valeric acid (2.0 g/L)	4.8	34	1.6	47	0.3	0.14	[60]
*Methylobacterium organophilum* NCIB 11278 Methanol (4.0 g/L) Valeric aid (0.5 g/L)	2.5	50	1.3	10	0.1	0.25	[77]
*Burkholderis* sp. IS-01 Gluconate (20.0 g/L) Levulinic acid (12.5 g/L)	5.9	62	3.7	87	3.2	0.25	[67]
*C. necator* KHB-8862 Fructose syrup (20.0 g/L) Levulinic acid (1.0 g/L, initial and 3 times feeding)	8.6	84	7.2	28	2.0	0.50	[66]
*C. necator* H16 Fructose (20.0 g/L) Levulinic acid (3.5 g/L)	7.3	48	3.5	16	0.6	0.16	[11]
*Hydrogenophaga pseudoflava* DSM 1034 Whey permeate (47 mL/L) Levulinic acid (1.0 g/L)	4.5	49	2.2	45	1.0	1.00	[68]
Conjugate bases of organic acids							
*Caldimonas taiwanensis* Sugars (1.5%) Valerate (0.5 g/L)	1.6–4.1	42–67	0.8–2.1	10–13	0.1–0.2	0.16–0.49	[78]
*Methylocystis* dominated mixed culture Methane gas (repeating 48 h fed-batch cycle) Valerate (0.4 g/L)	1.5	30	0.5	39	0.2	0.45	[79]
Sodium salts of organic acids							
*Azohydromonas lata* Rice wastewater (21 g/L) Sodium acetate (10 g/L)	5.0	32	1.6	6	0.1	0.01	[80]
*Corynebacterium glutamicum* ATCC13869 transformant ^A^ Sodium propionate (1.0 g/L)	-	31	-	28	-	-	[81]
*C. necator* H16 Sodium acetate (0–20 g/L) Sodium propionate (0–20 g/L)	0.3–0.7	12–56	Trace	0–45	Trace	-	[82]
*C. necator* PHB^−^4 ^C^ Palm kernel oil (5.0 g/L) Sodium propionate (5.0 g/L)	3.0	30	0.9	12	0.1	0.02	[83]
*Herbaspirillum seropedicae* Z69Prp ^D^ Glucose (7.0 g/L) Sodium propionate (0.5 g/L)	2.4	37	0.9	14	0.1	0.25	[84]
*C. necator* H16 Plant oils (5.0 g/L) Sodium valerate (5.0 g/L)	4.1–6.1	64–89	2.1–5.4	3–14	0.1–0.9	0.03–0.17	[85]
*C. necator* PHB^−^4 ^C^ Palm kernel oil (5.0 g/L) Sodium valerate (1.0 g/L)	4.2	52	2.2	6	0.1	0.13	[83]
*Methylocystis parvus* OBB3 Methane gas (75 mL) Sodium valerate (1.0 g/L)	-	-	0.3	-	0.2	0.20	[86]
Alkyl alcohols							
*C. necator* H16 Waste rapeseed oil (20.0 g/L) 1-propanol (8.0 g/L)	14.7	80	11.7	9	1.1	0.14	[13]
*Erwinia* sp. USMI-20 Palm oil (4.6 g/L) 1-propanol (2.3 g/L)	5.4	50	2.7	6	0.2	0.07	[60]
*C. malaysiensis* USMAA2-4 Oleic acid (6.5 g/L) 1-pentanol (0.9 g/L)	5.1	40	2.1	8	0.2	0.22	[87]
*C. malaysiensis* USMAA2-4_ABH16_^B^ Palm olein (6.5 g/L) 1-pentanol (0.9 g/L)	5.4	69	3.7	7	0.3	0.33	[87]
*C. malaysiensis* USMAA1020 Oleic acid (6.5 g/L) 1-pentanol (1.3 g/L)	-	76	-	10	-	-	[88]
*Erwinia* sp. USMI-20 Palm oil (4.6 g/L) 1-pentanol (1.4 g/L)	4.8	62	3.0	20	0.6	0.43	[60]
*Massilia haematophila* UMTKB-2 Glucose (16.0 g/L) 1-pentanol (1 g/L)	-	-	5.0	7	0.4	0.40	[89]
*Methylobacterium extorquens* G10 Methanol (fractional supply by 5–20 mL) 1-pentanol (fractional supply by 2%–20% v/v methanol)	25–40	30–45	7.5–18.0	14–50	2.5–4.5	-	[90]
*Methylocystis* sp. WRRC1 Methane gas (75 mL) 1-pentanol (1.0 g/L)	-	-	0.3	-	0.2	0.17	[86]
*Methyloligella halotolerans* C2 Methanol (5–20 mL fractional supply) 1-pentanol (fractional supply by 5–15% *v*/*v* methanol)	-	49–98	-	2–51	-	-	[91]
*P. denitrificans* ATCC 17741 1-pentanol (maintained at 1.6 g/L)	6.8	18	1.2	100	1.2	-	[14]
Mixed precursors							
*C. necator* DSM 545 Levulinic acid (1.0 g/L) Sodium propionate (2.5 g/L)	1.0	33	0.3	73	0.2	0.24	[65]
*C. necator* DSM 545 Levulinic acid (1.0 g/L) Sodium propionate (1.0 g/L)	0.5	19	0.1	78	Trace	-	[10]
*H. pseudoflava* DSM 1034 Whey permeate (47.0 mL/L) Levulinic acid (0.5 g/L, initial and 3 times feeding) Sodium valerate (1.0 g/L, initial and 3 times feeding)	6.6	67	4.4	55	2.4	0.43	[68]

Only the most promising condition was included for studies involving multiple cultivation conditions. Trace (concentration < 0.1 g/L). ^A^ *C. glutamicum* ATCC13869 transformant harboring *C. necator phaCAB*_Re_ genes. ^B^ *C. malaysiensis* USMAA2-4 transformant harboring *C. necator* H16 *lipAB* genes. ^C^ *C. necator* mutant with P(3HB)-negative phenotype [92]. ^D^ *H. seropedicae* Z69 with the 2-methylcitrate synthase (*PrpC*) gene eliminated.

## Abbreviations

3HB	3-hydroxybutyrate
3HV	3-hydroxyvalerate
*α*-P(3HB)	Synthetic atactic poly(3-hydroxybutyrate)
Ag/BSA	Bovine serum albumin capped silver
AS	Ascorbic acid
CNC	Cellulose nanocrystals
CNT	Carbon nanotubes
DCP	Dicumyl peroxide
HA	Hydroxyapatite
HDPE	High-density polyethylene
LDPE	Low-density polyethylene
LLDPE	Linear low-density polyethylene
MAT	Organophilic attapulgite
mPEG	Monomethoxy poly(ethylene glycol)
NA	Not available
P(3HB)	Poly(3-hydroxybutyrate)
P(3HV)	Poly(3-hydroxyvalerate)
P(3HB-*co*-3HV)	Poly(3-hydroxybutyrate-*co*-3-hydroxyvalerate)
PBAT	Poly(butylene adipate-*co*-terephthalate)
PBS	Poly(butylene succinate)
PCL	Poly(*ε*-caprolactone)
PDLLA	Poly(d,l-lactide)
PEG	Poly(ethylene glycol)
PHA	Polyhydroxyalkanoates
PHEMA	Poly(2-hydroxyl ethyl methacrylate)
PLA	Poly(lactic acid)
PP	Polypropylene
PPC	Poly(propylene carbonate)
Ref.	References
SBM	Sleeping beauty mutase
